# Fostering equity in precision health through diverse 3D facial data

**DOI:** 10.3389/fmedt.2026.1717535

**Published:** 2026-02-19

**Authors:** Saumya Jamuar, Richard Palmer, Zi Qiang Teo, Stuart Lee, Petra Helmholz, Shermaine Chan, Gareth Baynam

**Affiliations:** 1 Genetics Service, KK Women’s and Children’s Hospital, Singapore, Singapore; 2 SingHealth Duke-NUS Institute of Precision Medicine, Singapore, Singapore; 3 SingHealth Duke-NUS Genomic Medicine Centre, Singapore, Singapore; 4 School of Earth and Planetary Sciences, Curtin University, Perth, WA, Australia; 5 Takeda Pharmaceuticals International AG, Singapore Branch (at the Time of Manuscript Development), Singapore, Singapore; 6 Takeda Pharmaceuticals (Asia Pacific) Pte Ltd, Singapore, Singapore; 7 KK Research Centre, KK Women’s and Children’s Hospital, Singapore, Singapore; 8 Rare Care Centre, Perth Children’s Hospital, Perth, WA, Australia; 9 Western Australian Register of Developmental Anomalies, King Edward Memorial Hospital, Perth, WA, Australia

**Keywords:** 3D facial imaging, facial diversities, genetic ancestries, health equity, precision health

## Abstract

**Introduction:**

The promise of precision medicine lies in its ability to provide greater diagnostic accuracy and customized therapy by filtering out patients less likely to benefit from it. Our study focuses on the importance of reducing uncertainty in interpretation of individuals 3D facial data to support more equitable precision medicine applications. The Human Genome Project and subsequent advances in sequencing have led to the creation of vast genetic datasets, predominantly representing individuals of European origin. However, there is a significant underrepresentation of individuals of African, Asian, and Indigenous ancestries.

**Methods:**

The study involved 1,218 participants from various genetic ancestries backgrounds, with a focus on the paediatric population of Chinese genetic ancestry. The study subjects underwent 3D facial photogrammetry in outpatient department setting and with the aid of Cliniface software growth curves were obtained to produce reference statistics of 3D facial norms.

**Results:**

The results showed measurable and distinct facial differences in children with Chinese genetic ancestry when compared with other groups representing different genetic ancestries highlighting the need for population diversity and inclusion enrichment in genetic databases. Also, these facial differences and markers are uniquely poised to be correlated in clinic as disease specific digital biomarkers with further investigation and validation in conditions such as hereditary angioedema.

**Discussion:**

The study underscores the importance of creating larger datasets involving more diverse genetic ancestry groups to enhance the evidence base for advanced and equitable disease diagnosis, treatment monitoring, prognostication and customized drug development.

## 1 Introduction

The promise of precision medicine is embedded in greater diagnostic accuracy and customized therapy while filtering out patients who are less likely to benefit from it. Appropriate phenotypic and genotypic interpretation forms the core of precision medicine applications (1). Therefore, reducing the uncertainty in individual genetic information interpretation becomes extremely important.

The Human Genome Project international consortium's human genetic sequencing was a watershed moment (2, 3). Since then, technical advances in sequencing and a drastic reduction in the cost of sequencing have fueled the creation of vast numbers and sizes of genetic datasets (4). Biobank Japan project (5), WISDOM (Women Informed to Screen Depending on Measures of Risk) (6), and UK Biobank (7) are some of the key projects we would want to highlight. Individuals of European origin find major representation in the UK or US-led genetic studies. Similarly, Biobank Japan had participants of almost all Japanese origin.

Thousands of genetic risk variants and their biological function have been identified through 3,700 genome-wide association studies (GWAS) since the first human genome sequencing in 2003 (8–10). GWAS conducted between 2005 and 2018 (3,639 studies; 3,508 traits), and the scientometric review revealed an exponential increase in the size of samples, discovery rates, and studied traits. Upon longitudinal examination, fluctuating ancestral diversity patterns exist, but European Ancestry (88% in 2017) predominates with 72% of discoveries. Participants were recruited from three countries (US, UK, Iceland), and NIH funded 85% of these studies (11). Individuals of African or Asian ancestry are grossly underrepresented in proportion to the population they hail from. Groups, such as Indigenous people in the USA, Canada, the Caribbean, Polynesia, Australia, etc., have yet to find inclusion in these studies (4). Beyond the lack of knowledge on the genetic architecture of non-European people, it is of concern that medical decisions grounded on the analysis of Eurocentric genetic scores may amplify healthcare disparities.

Population diversity, inclusion enrichment in genetic databases, and evaluation of genetic scores in combination with other disease factors are needed to enable a greater equitable impact and translate the true potential of precision medicine. The Human Heredity and Health in Africa (H3Africa) (12), Global Biobank Meta-Analysis Initiative (GBMI) (13), GenomeAsia100K (14), −1,000 (15) are some of the notable efforts to foster inclusion and bring the individuals from population groups that have been neglected in the past.

Definable traits such as morphology, biochemical, physical, etc., in individuals or larger groups involve phenomic measurement. Phenomics is an intricate and new era of science that sets out to measure and study phenomes at scale (16), for application at the phenotypic level or in combination with genomic data. These traits evolve from a complex interplay of genomic, environmental, dietary patterns, microbiome, etc. The application of imaging techniques and analysis of human phenomics results in Phenomic Imaging (PI). The PI has several advantages as it is scalable, non-invasive, replicable, simpler, and captures robust spatiotemporal data that furnish photographs of lively and immediate changes in the intended or targeted organs (17, 18).

Singapore, an island nation with a population comprising different genetic ancestries, has initiated Project SG100K to map 100,000 participants' genomes. The database is expected to be one of Asia's leading reference genome databases since the nation's genetic ancestry diversity represents more than 80 percent of Asia's diversity (19).

In this study, we present reference statistics of 58 facial measurement norms from 3D facial images captured from a large sample of Singapore's majority Chinese ethnic population (*N* = 879). Deeply precise diagnostic usages are being applied by deploying 3D facial analysis for Rare Diseases (RDs) with facial dysmorphic features (20). Facial diagnostic signatures are being unlocked which were otherwise undetected and unnoticed earlier such as fibrodysplasia ossificans progressiva (21). Also, facial signatures identified with 3D facial imaging can be utilized for identifying language delays and conditions such as Angelman-like syndromes distinguished by severe speech impairment and underlying facial features (22). By deploying 3D photography, we aim to differentiate and stratify facial features to enhance the evidence base for advanced and equitable disease diagnosis and treatment monitoring.

## 2 Methods

This was an observational cross-sectional study to characterize and quantify the facial characteristics of the Singaporean population using 3D photogrammetry and analysis using the Cliniface software application. Cliniface is free-to-use software that uses 3D facial images to visualize, analyze, examine anthropometrics, and detect dysmorphological characteristics. Irrespective of the image generation mechanism, the software enables and facilitates the visualization and investigation of 3D images of patients or study participants. Interactive viewership of 3D facial images, obtaining more than 50 distinguishable measurements, and generating a report if clinically significant specific phenotypic facial traits from more than 40 different types are present in the database are some of the distinct functions of Cliniface (23). The software was chosen due to its abilities which include:

- Viewing facial details at a sub-millimeter level of accuracy (depending on the precision of the image capture hardware used),
- Accurate placement of clinically salient landmarks on the 3D facial surface,
- Automatic extraction of 58 different facial measurements of different types (including angles, distances, depth, and asymmetry),
- Exporting of facial landmarks and measurements in deidentified text formats,
- Integrating custom statistical growth data for clinical evaluation of individuals.

The sample used to estimate this population consisted of 1,218 participants of diverse genetic ancestries—screened to exclude those with atypical facial features due to pre-existing conditions or past medical history. Table 1 shows the distribution of all genetic ancestries sampled in this study.

**Table 1:** Genetic ancestry-specific distribution of study subjects.

Genetic ancestry	Count	Percentage
Chinese	879	72.2
Malay	179	14.7
Indian	96	7.9
Filipino	13	1.1
Nepalese	6	0.5
Caucasian	5	0.4
Sri Lankan	4	0.3
Myanmar	3	0.2
Arab	3	0.2
Bangladeshi	3	0.2
Pakistani	3	0.2
Vietnamese	3	0.2
Boyanese	3	0.2
African	3	0.2
Indonesian	3	0.2
Javanese	2	0.2
Korean	2	0.2
Burmese	2	0.2
Chinese-thai	1	0.1
Unknown	1	0.1
Singhalese-chinese	1	0.1
Jew-chinese	1	0.1
Chinese-burmese	1	0.1
Chinese-japanese	1	0.1
	1,218	100.0%

**Figure 1:**
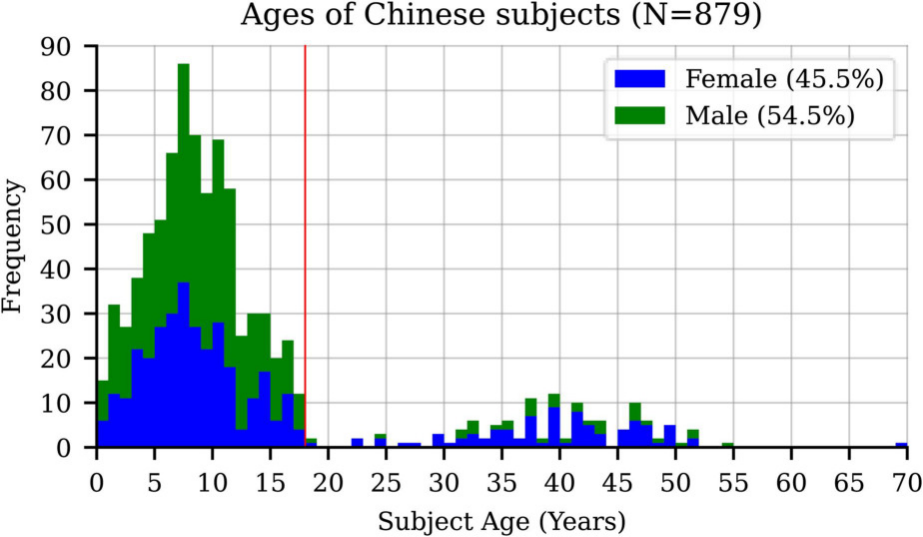
Age and sex-based representation in the study group.

After capturing facial images of the 1,218 participants, the group was narrowed down to just those of Chinese genetic ancestry (who comprise most of Singapore's population). Figure 1 shows the age group-specific representation of this group. Of the 879 Chinese subjects, 758 were younger than 18 years old, hence our analysis focused primarily on pediatric age groups. Age specific distribution is depicted in the Supplementary Table.

A 3D facial image of each participant was taken by a member of the study team in Singapore using the Vectra M3 camera system. The process took no more than 20 min for each participant. To reduce the potential for partial occlusion of the face, participants were asked to adjust clothing around the neck/chin and to tie back hair where necessary before their image was captured. Each participant's 3D facial image was photogrammetrically stitched together from three simultaneously captured photographs taken of the front and both sides of the face in accordance with the fixed geometry of the Vectra M3 system's camera “pods”. The stitching process took approximately a minute per capture, and captures were repeated if the final stitched 3D image was found to be poor in quality. The final stitched 3D facial image of each participant covered the extents of the front of the face and forehead and under the chin as far back as the most anterior part of the ears. The image was then imported into Cliniface to map facial landmarks and to extract the 58 different measurements.

The resulting de-identified measurement data was transmitted to the Cliniface team in Western Australia (WA) whereupon it was collated, aggregated, and analyzed to generate the population-specific growth curves for all 58 of the facial measurements. Once generated, the growth curve summary statistics were incorporated back into Cliniface to allow for individualized assessments.

### 2.1 Study site

The study was conducted at KK Women's and Children's Hospital of Singapore between the 13th July 2021 and the 30th June 2024. Participants were recruited under the SingHealth Institutional Review Board approved project (CIRB 2021/2419). 3D facial photographs were taken with the Vectra M3 camera system (Canfield, USA). Deidentified summary data was extracted using Cliniface (Perth, Australia). Parental consent was obtained for all participants in the pediatric group.

Demographic data included age, gender, genetic ancestry of participant and biological parents, past medical or surgical history, including history of facial surgery or trauma (attached questionnaire). Participants for control images were recruited primarily from the Paediatric outpatient clinics at KK Women's and Children's Hospital, Singapore. Where possible, parents and siblings of the index participant were recruited as well.

Participants were recruited through convenience sampling of hospital visitors resulting in proportional stratified sampling of the different ethnic groups. Recruitment was tracked to ensure uniform coverage of the target age range (0–18 years) and equal gender balance.

**Figure 2:**
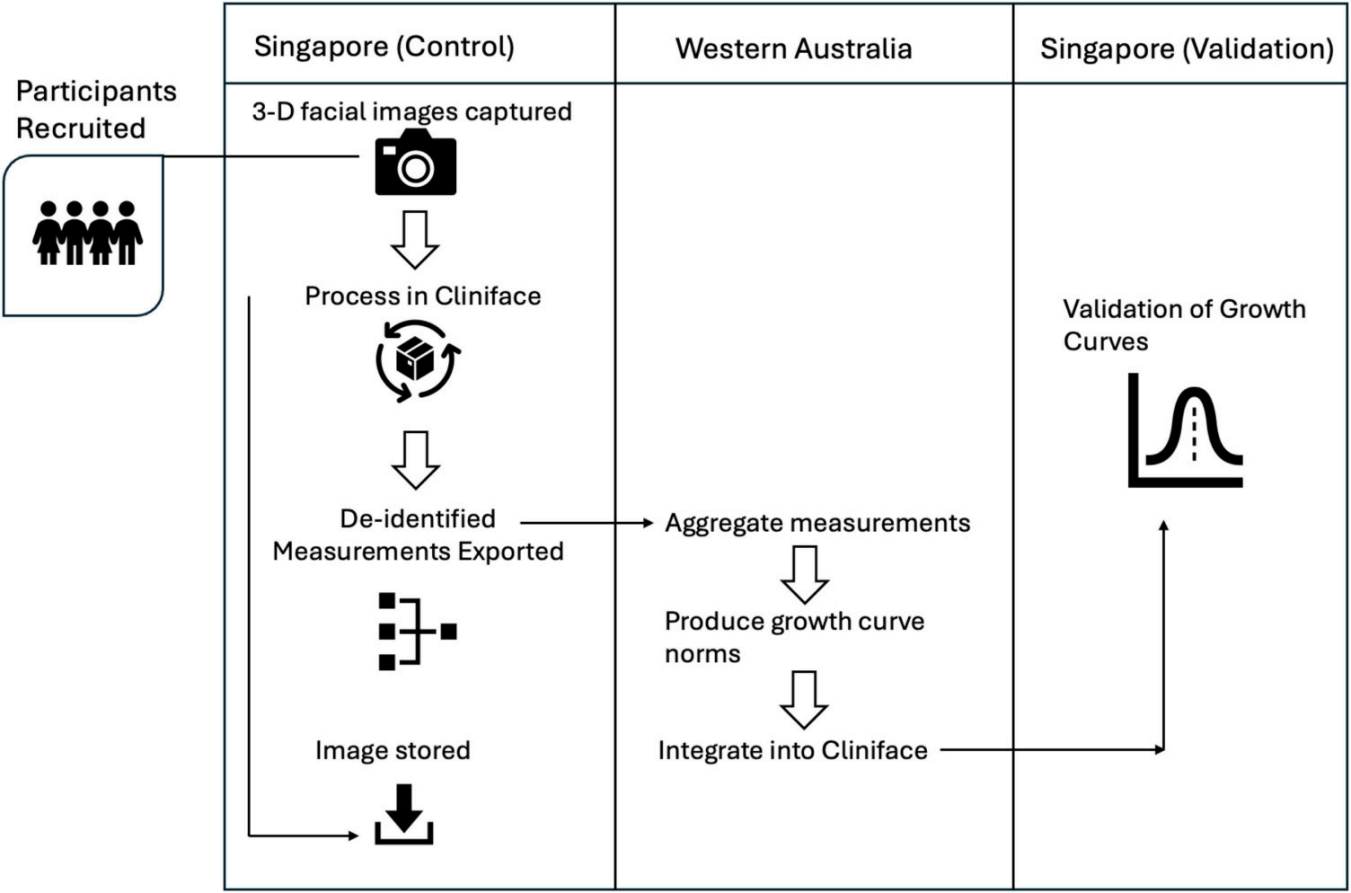
Study design flow chart.

3D facial images were collected and other metadata of analytic relevance (e.g., age, sex, and genetic ancestry) was provided by the imaging/processing clinician. All 3D facial images were retained within local jurisdictions (i.e., Singapore). Metadata was collected using a research coordinator-administered questionnaire. Participation was one-time and involved obtaining consent, collecting relevant health information, and performing 3D photography. The flow chart of Figure 2 describes the study design.

### 2.2 Ethical considerations

Participation in this study was voluntary, and participants were advised that they could withdraw at any time. However, any data collected until the withdrawal was kept and analyzed. No compensation was provided.

### 2.3 Data extraction

Study members responsible for landmark placement and measurement extraction were first trained to confirm the placement of facial landmarks suggested by Cliniface via its automatic landmark detection mechanism which seeks to accurately place facial landmarks in their correct positions before allowing manual adjustment and confirmation of those landmark positions (23). This training was conducted by more experienced study members. A selection of out-of-sample training images were used for this purpose, and these were evaluated against pre-defined ground-truth placement of the facial landmarks to determine landmark placement accuracy by the study members. If large discrepancies were seen (more than 5 mm), further training was provided and the study member was reassessed until their placements were within tolerance. Study members were only allowed to confirm landmark placement within the study dataset's images after passing this training stage. In all cases, the landmark positions on the study dataset's images were cross-checked by a second more experienced study member.

**Figure 3:**
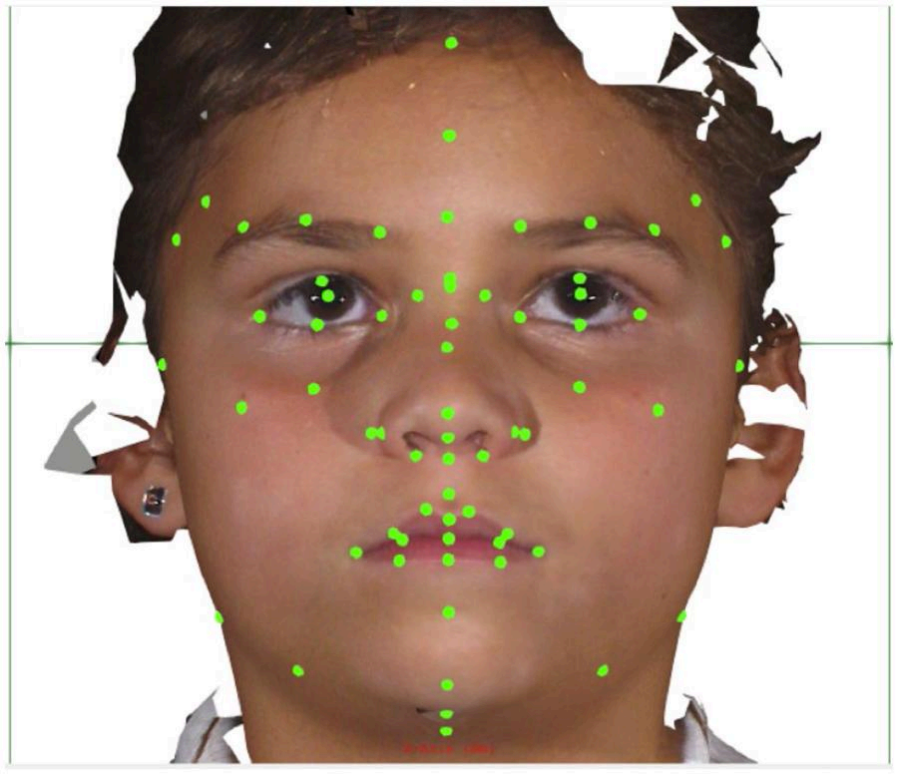
A screenshot from cliniface showing the facial landmarks (19 medial and 25 bilateral) as yellow dots overlayed on one of the sample 3D facial image used to help train study members in landmark placement (image by cliniface).

In the case of some images, certain landmarks were deemed unusable due to localized quality issues encountered during the photogrammetric generation of the 3D facial surface. In other instances, occlusions due to hair or clothing around the facial boundary prevented precise extraction of facial landmarks in those regions. In such cases, the specific landmarks (and their related measurements) were noted for exclusion during later analysis so that other landmarks and their related measurements in that facial image could still be used. All facial measurements were derived from the placements of the facial landmarks. Figure 3 shows the Cliniface program with the full set of facial landmarks (19 medial and 25 bilateral) as yellow dots overlayed on one of Cliniface's included example images.

### 2.4 Data analysis

De-identified data containing the 58 facial measurements, subject age, and subject sex were collated from all images and transmitted to the study team in Western Australia (WA). The data were then aggregated and analyzed to generate growth curves for every facial measurement. Characterization of reference range variation with sufficient power to detect half a standard deviation in measurement with 95% confidence was targeted. Checked against an estimate of variance for a typical facial measurement (intercanthal width) in individuals of European genetic ancestry, a 5% significance level with 80% power was found to require images from a minimum of 30 individuals in a single age/sex band (with an assumed fall-out/rejection rate of 15%). Regression modelling of measurements over the whole of the sampled age range was undertaken due to the logistical difficulty of ensuring consistent sampling density across all age bands.

The regression model used for each measurement was chosen by minimizing the residual sum of squared errors from the best fit of each model for each measurement's data points. A model for each measurement was chosen from five different candidates:

1. an exponential decay function in three parameters of form: y = a(−e^(1− bx) + c),
2. a linear function of form: y = ax + b,
3. a quadratic of form: y = ax^2 + bx + c,
4. a degree 3 polynomial of form: y = ax^3 + bx^2 + cx + d, and
5. a degree 4 polynomial of form: y = ax^4 + bx^3 + cx^2 + dx + e.

Initial modelling of the measurement data was performed to identify outliers in the data (defined as more than three standard deviations from the modelled mean). The identity of these outlier images and the relevant landmarks was communicated to the study team in Singapore, who then reappraised the positions of those landmarks in the respective images before sending back the updated data for reanalysis. Regression modelling was then performed again against the reaggregated data to produce a growth curve for each measurement and for each of the sexes. Modelling using an exponential decay function resulted in the best fit to the data in most cases. This aligns with previous research of comparative model-fitting in craniofacial measurements where models with an exponential component to drive trajectories of growth through early childhood tend to work very well across different cohorts (24–26).

Standard deviations and 95% confidence intervals were also calculated. These statistics varied with age due to the non-uniformity of the sampled age distribution. However, the overall variance in these confidence intervals was constrained due to regression fitting over the whole dataset (including older participants where growth had plateaued) rather than just individual age bands. The final growth curve statistics for each measurement were incorporated into Cliniface to allow for atypical facial trait detection within the Chinese population at an individual level.

## 3 Results

After incorporating the newly generated reference statistics for the Chinese population into Cliniface, among 8 new (out-of-sample) individuals of Chinese ancestry, the median number of dysmorphic features identified per patient by Cliniface reduced from 5.5 (range 3–9) to 1 (range 0–3). This reduction was specifically due to Cliniface's comparison of the facial measurements from these individuals to the newly introduced Chinese reference range statistics instead of the pre-existing European derived statistics. In the case of two individuals of Malay genetic ancestry, a reduction in the number of phenotypic traits was also seen suggesting that the Malay facial phenotype more closely resembles the Chinese rather than the European facial phenotype. A reduction was also observed in an individual of Filipino genetic ancestry when comparing to the new Chinese reference range in preference to the European derived statistics. Interestingly, a reduction was not seen in two individuals of Indian and Pakistani genetic ancestry—though the type of atypical facial traits identified did shift. While limited in the number of individuals assessed—and further investigation is warranted—this result does suggest a need to build distinct reference ranges for the South Asian population. These results are summarized in Table 2 and the full breakdown of identified traits per individual is provided in the supplementary material.

**Table 2:** Change in the number of human phenotype ontology (HPO) terms identified by cliniface before the introduction of the new reference range, and after introducing the new reference range derived from individuals of Chinese genetic ancestry.

Genetic Ancestry	# Individuals	Median # HPO Terms	
		Before	After
Chinese	8	5.5	1.0
Malay	2	2.5	0.5
Filipino	1	4.0	0.0
Indian	1	3.0	3.0
Pakistani	1	5.0	6.0

**Figure 4:**
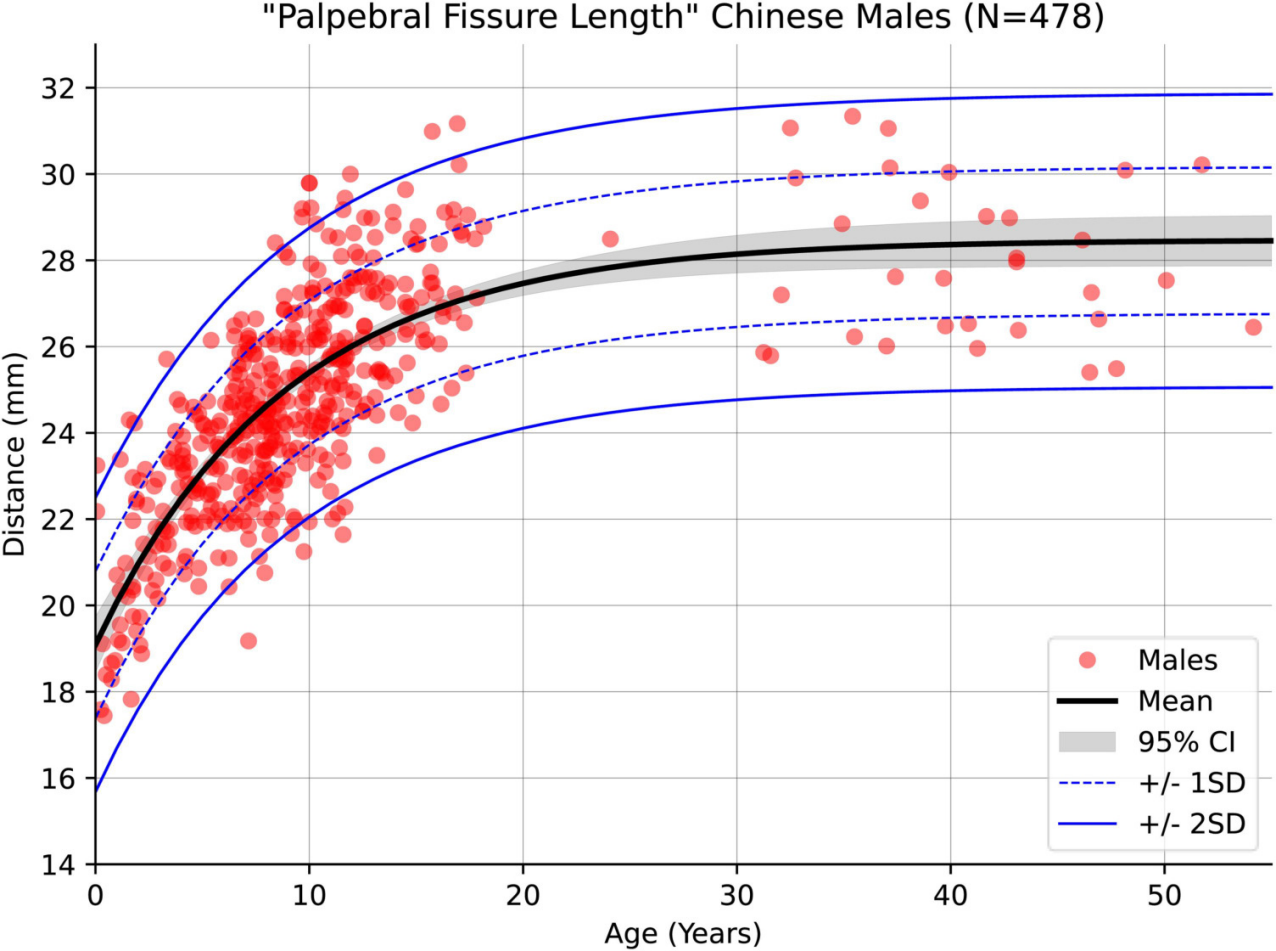
Male growth curve for palpebral fissure length.

**Figure 5:**
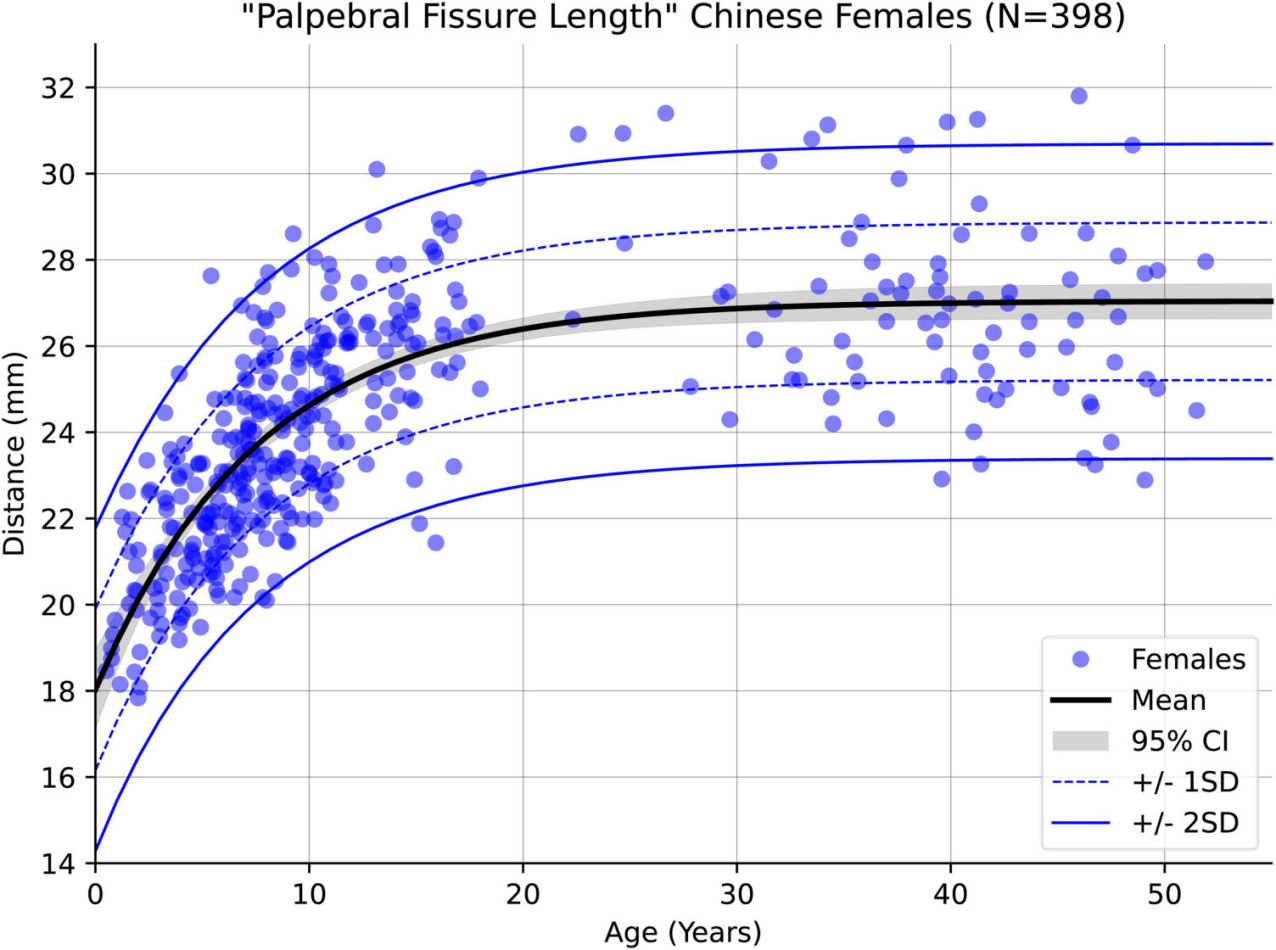
Female growth curve for palpebral fissure length.

Within the newly modelled Chinese reference range itself, distinct differences were observed between the sexes. One example is Palpebral Fissure Length (PFL) which showed a clear difference on average of approximately 1 mm with the distance in females being shorter on average across the whole of the sampled age range. Figures 4, 5 show the regression curves of this measurement for both sexes up to the age of 55 (the curves plateau further past this point). Explicit testing of this difference was not performed, however the 95% confidence intervals of the population mean for both sexes do not overlap likely indicating that the difference is statistically significant.

**Figure 6:**
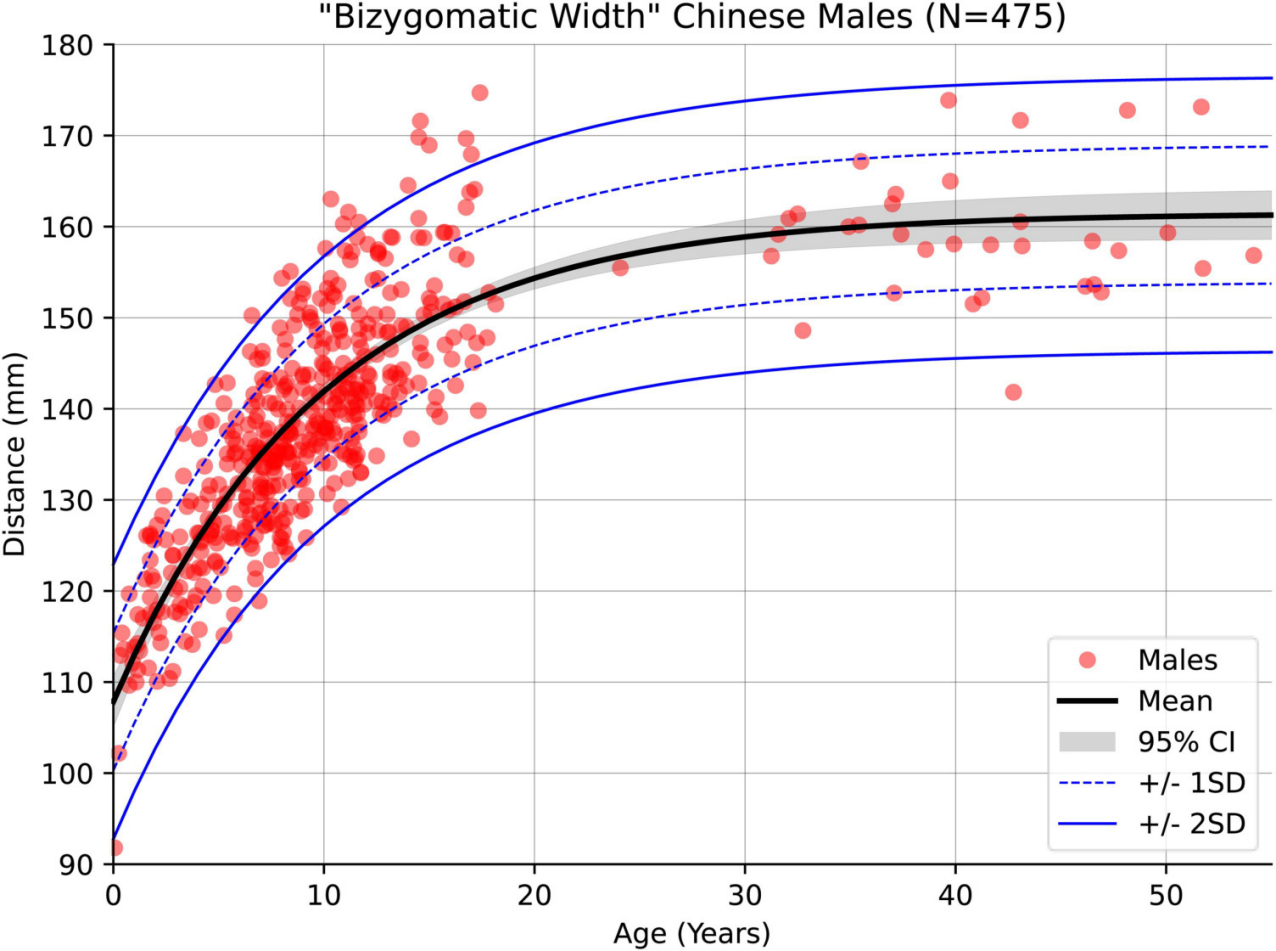
Male growth curve for bizygomatic width.

**Figure 7:**
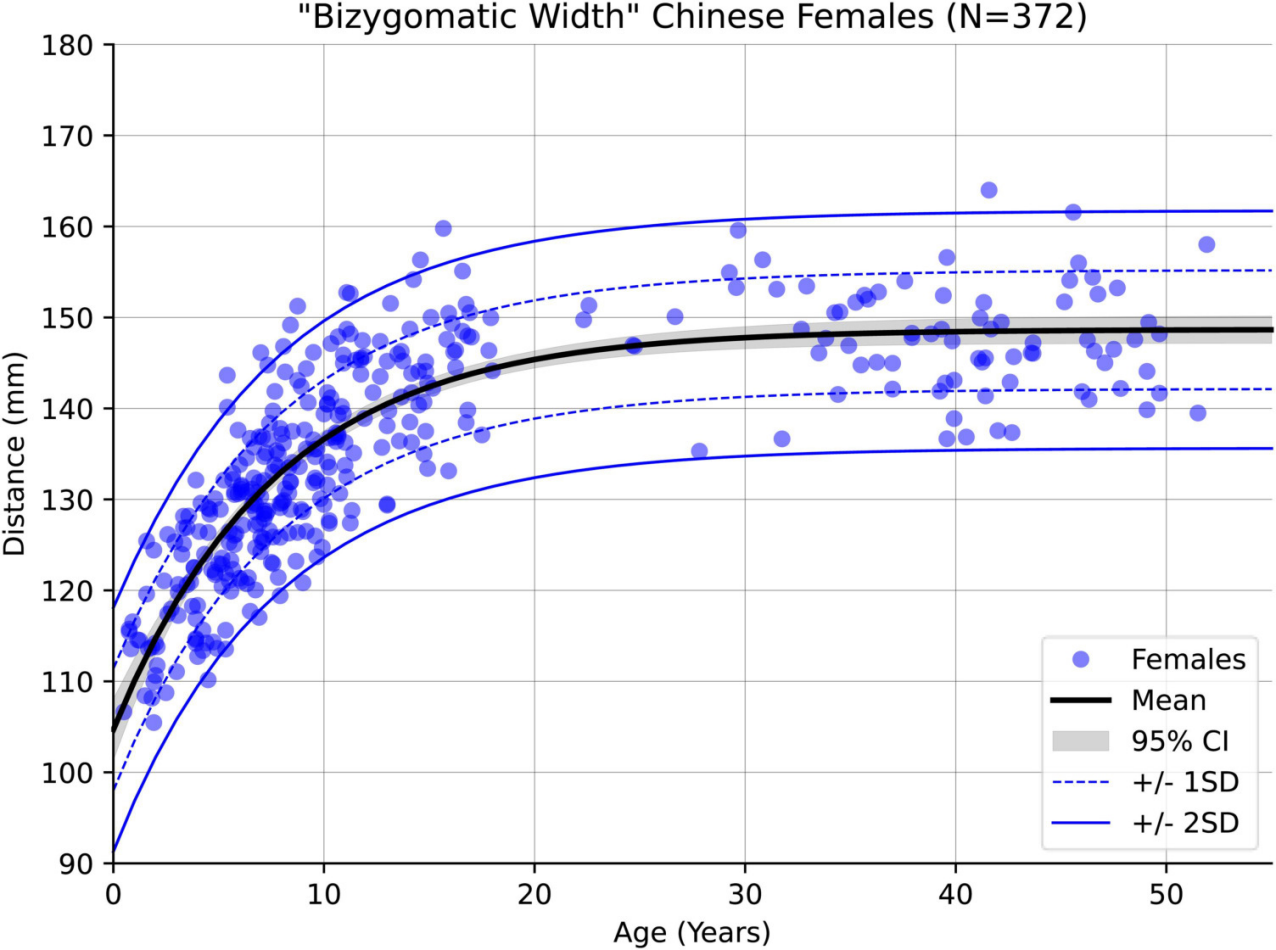
Female growth curve for bizygomatic width.

More pronounced sexual dimorphism was observed in the measurement of the bizygomatic width as Figures 6, 7 indicate which agrees with prior findings of this measurement (27).

Across the whole of the modelled age range, facial width was found to be significantly smaller in females than males. The mean difference being approximately 3.5 mm at age 5, rising to 5.2 mm at age 10 and 7.2 mm at age 15. The 95% confidence interval of these means is about ±0.5 mm at these ages in both males and females, showing that these results are statistically significant. Standard deviation remains consistent through childhood, being 6.8 mm (females) and 7.4 mm (males) at age 5, 6.6 mm (females) and 7.2 mm (males) at age 10, and 6.7 mm (females) and 7.6 mm (males) at age 15. Variance and the confidence interval for the regressed mean begin to widen slightly in the male cohort beyond the age of 20 due to the relatively decreased data density in that group, however this has little bearing on the significance of the results through childhood.

**Figure 8:**
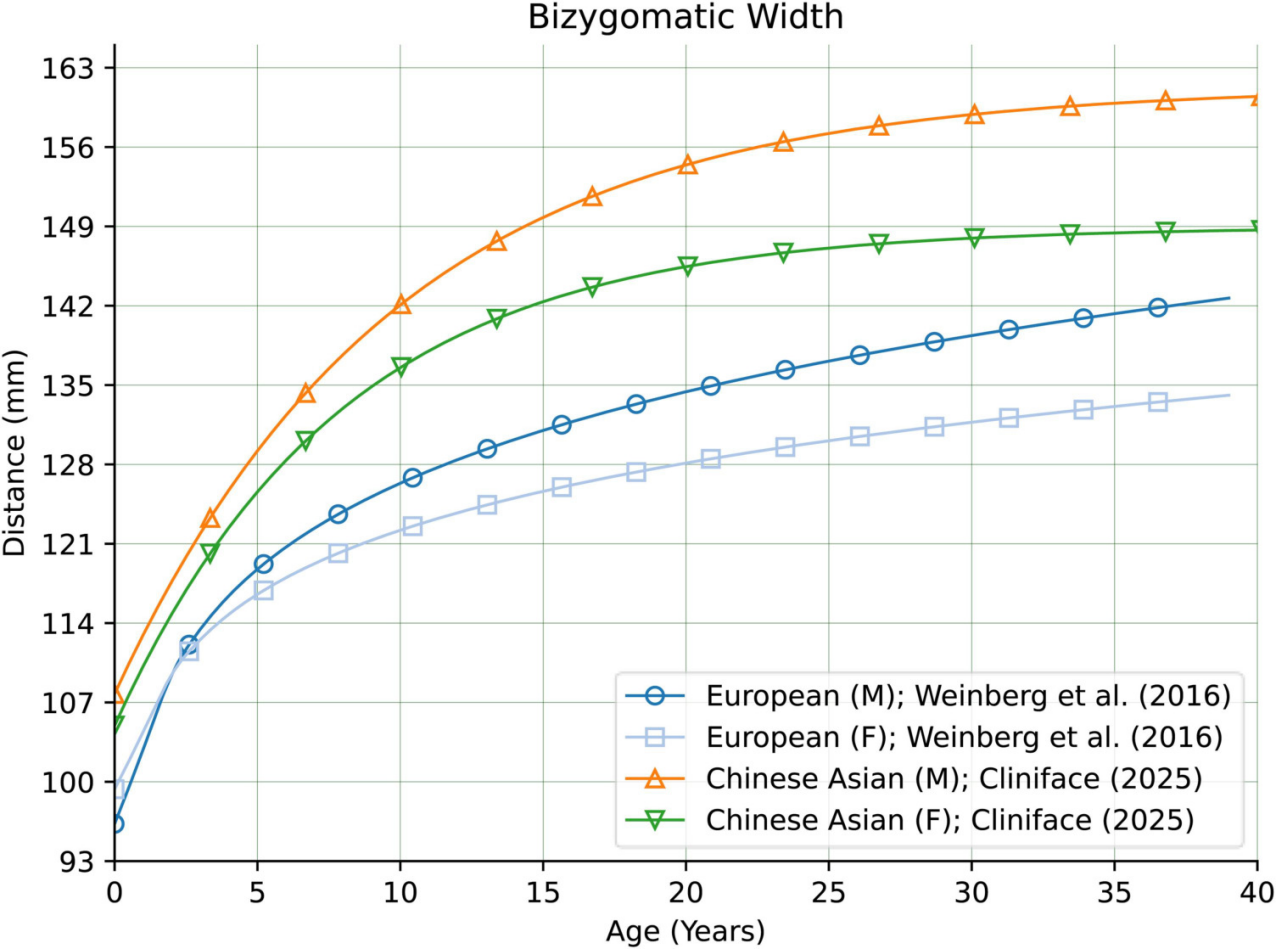
Comparison of growth curves for bizygomatic width comparing this work's estimated population means for males and females of Chinese genetic ancestry with males and females of European genetic ancestry.

When comparing our regressed means of facial width to the same measurement obtained from analysis of individuals with European genetic ancestry (28), it was found to be much larger in the Chinese group—at all ages. Figure 8 compares our Chinese male and female cohort regression means with the means of males and females in the European group. Note that the maximum age is truncated at 40 years for the sake of comparison with the European derived growth curves.

**Figure 9:**
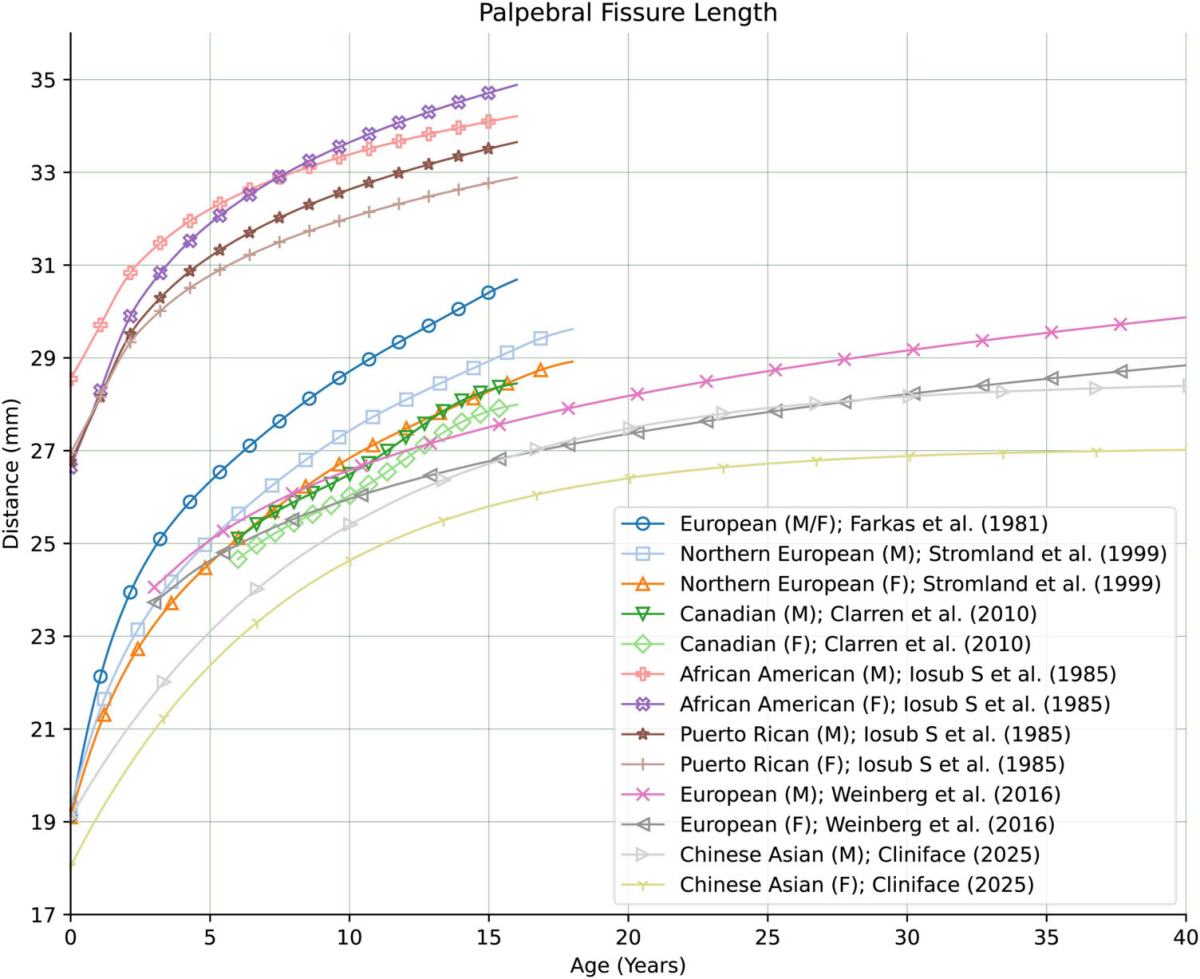
Comparison of growth curves for palpebral fissure length between different genetic ancestry groups. For the Chinese genetic ancestry group, the mean PFL in both males and females is smaller than in the other groups at all ages.

In comparing the regressed means of PFL from our Chinese cohort to those estimated from analysis of groups of different genetic ancestries (28–31), we found that our Chinese cohort was lower in both males and females than in the other groups (for matching sex). As seen in Figure 9, the magnitude of this difference is clearly significant when compared to the PFL curves for the African American, Canadian, European and Northern European groups—validating our efforts in creating a normative reference of facial measurements for people of Chinese genetic ancestry.

PFL was examined in normal children aged 1 month to 16 years, involving 340 participants, including 170 black and 170 Hispanic children, and revealed that black children generally exhibited longer palpebral fissures compared to white children and, in specific age groups, longer than their Hispanic counterparts (32). In a separate investigation in Canada, researchers analyzed a normative sample of 2,097 schoolchildren through photography. This study found that PFL increases with age, highlighting slight yet significant differences between boys and girls within each age group (30). Additionally, Strömland et al. conducted research to establish reference values for facial features in white Scandinavian children, using a sample of 613 healthy subjects (322 girls and 291 boys) aged 1 month to 18 years. The study also included nine children diagnosed with Fetal Alcohol Syndrome (FAS) aged 7–18 years. Using multivariate multiple regression analysis and facial feature curves, it was determined that children with FAS had significantly shorter PFL than their healthy peers (33).

**Figure 10:**
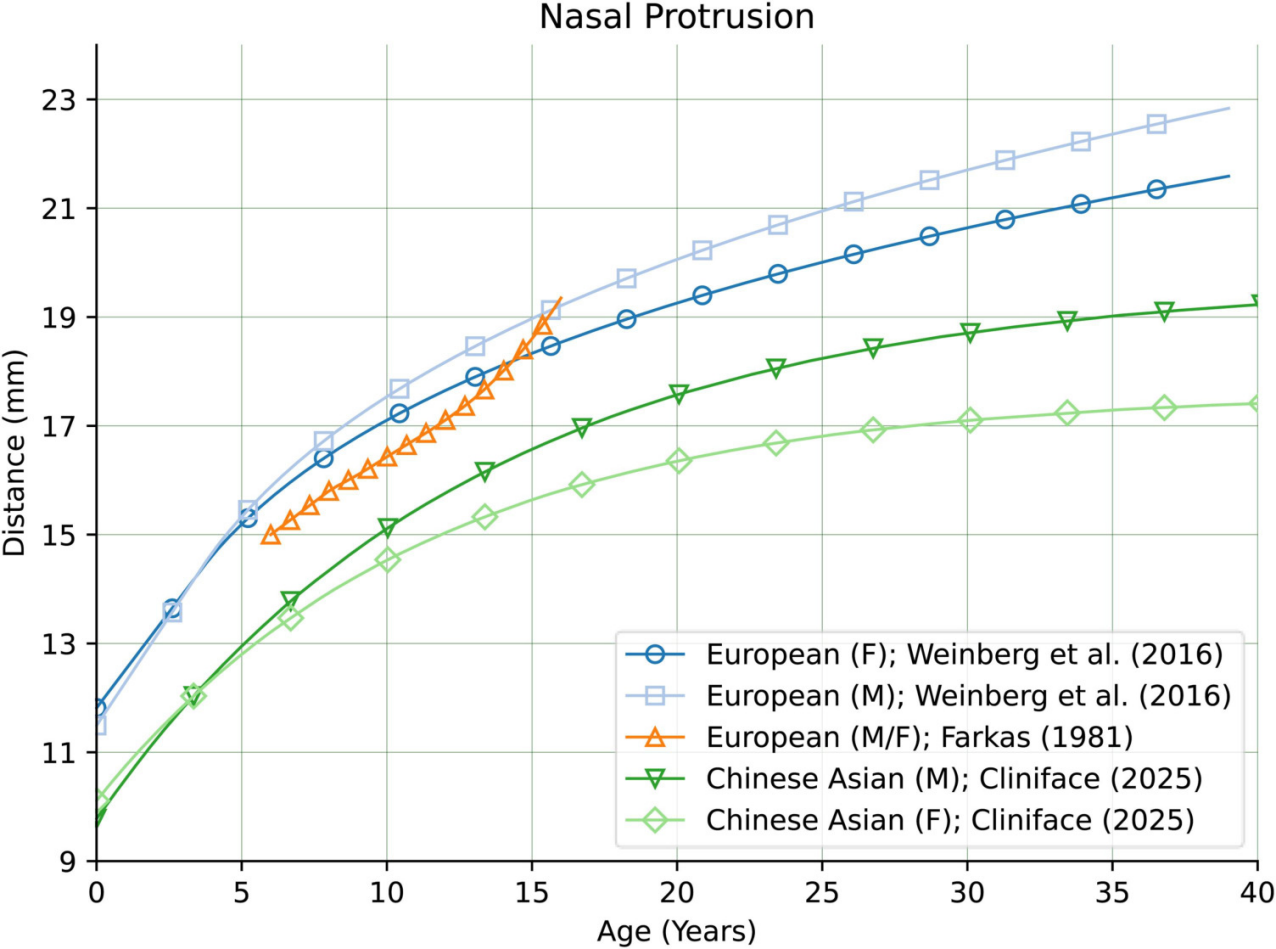
Nasal protrusion in males and females of Chinese vs. European genetic ancestry.

**Figure 11:**
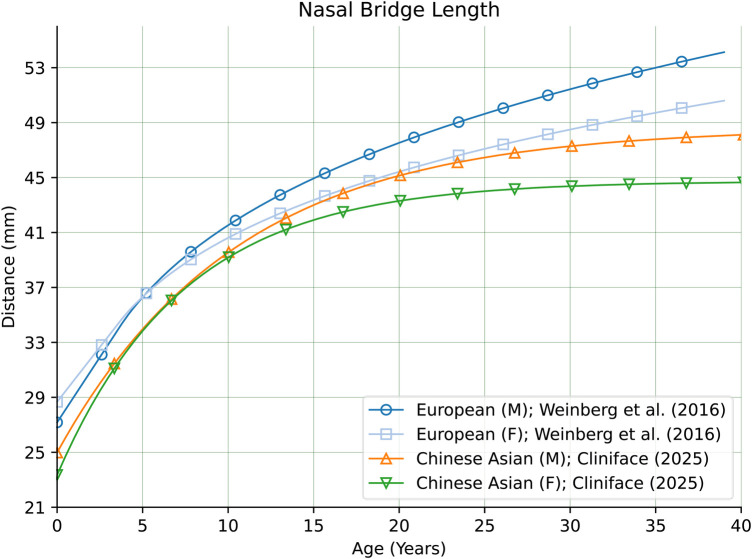
Nasal bridge length in males and females of Chinese vs. European genetic ancestry.

**Figure 12:**
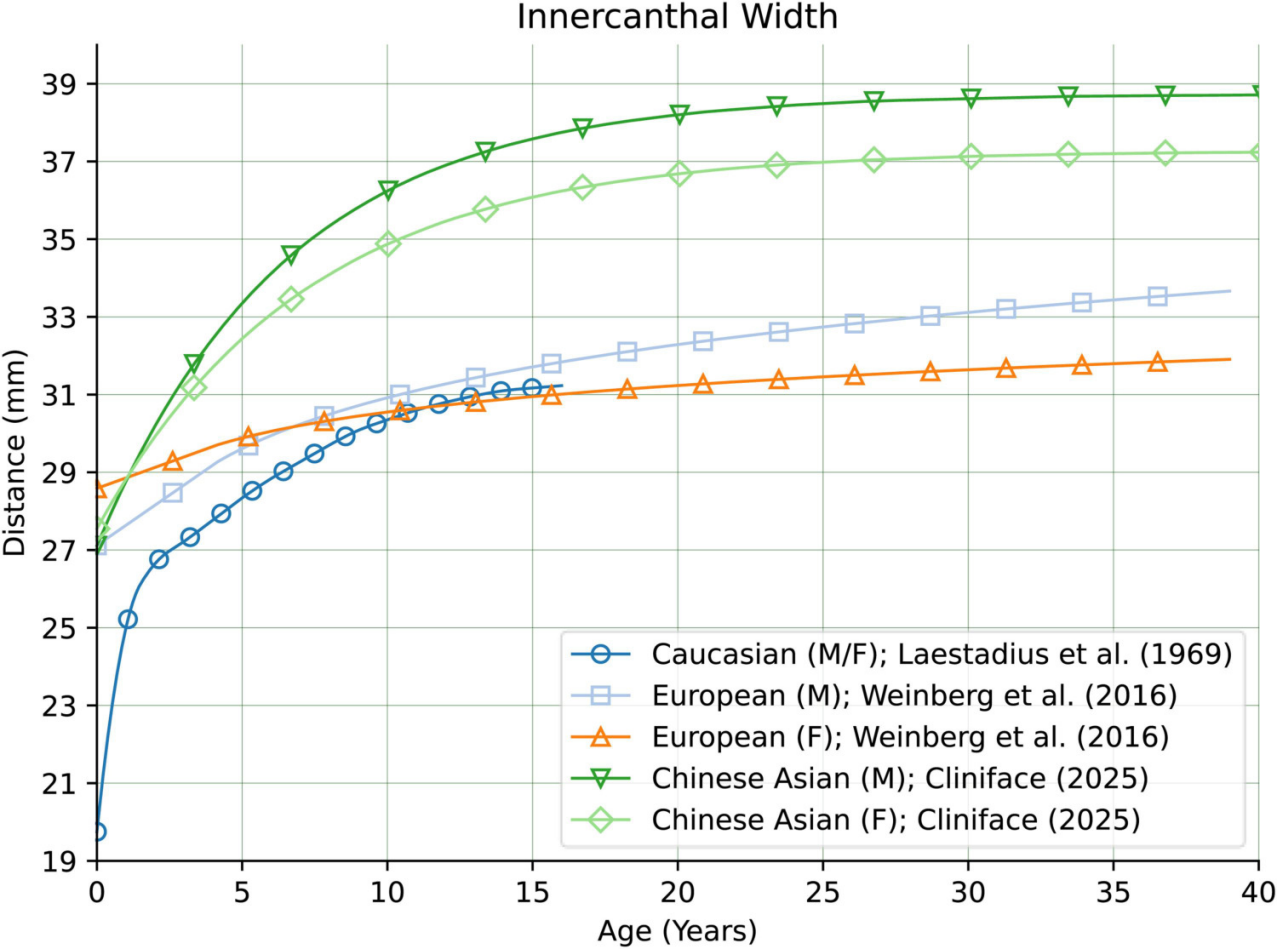
Innercanthal width in males and females of Chinese vs. European genetic ancestry.

There are also comparatively large differences in other facial features between our Chinese reference group and groups of primarily European genetic ancestry as seen in Figure 10 for Nasal Protrusion (28, 31), Figure 11 for Nasal Bridge Length (28), and Figure 12 for Innercanthal Length (28, 34).

**Figure 13:**
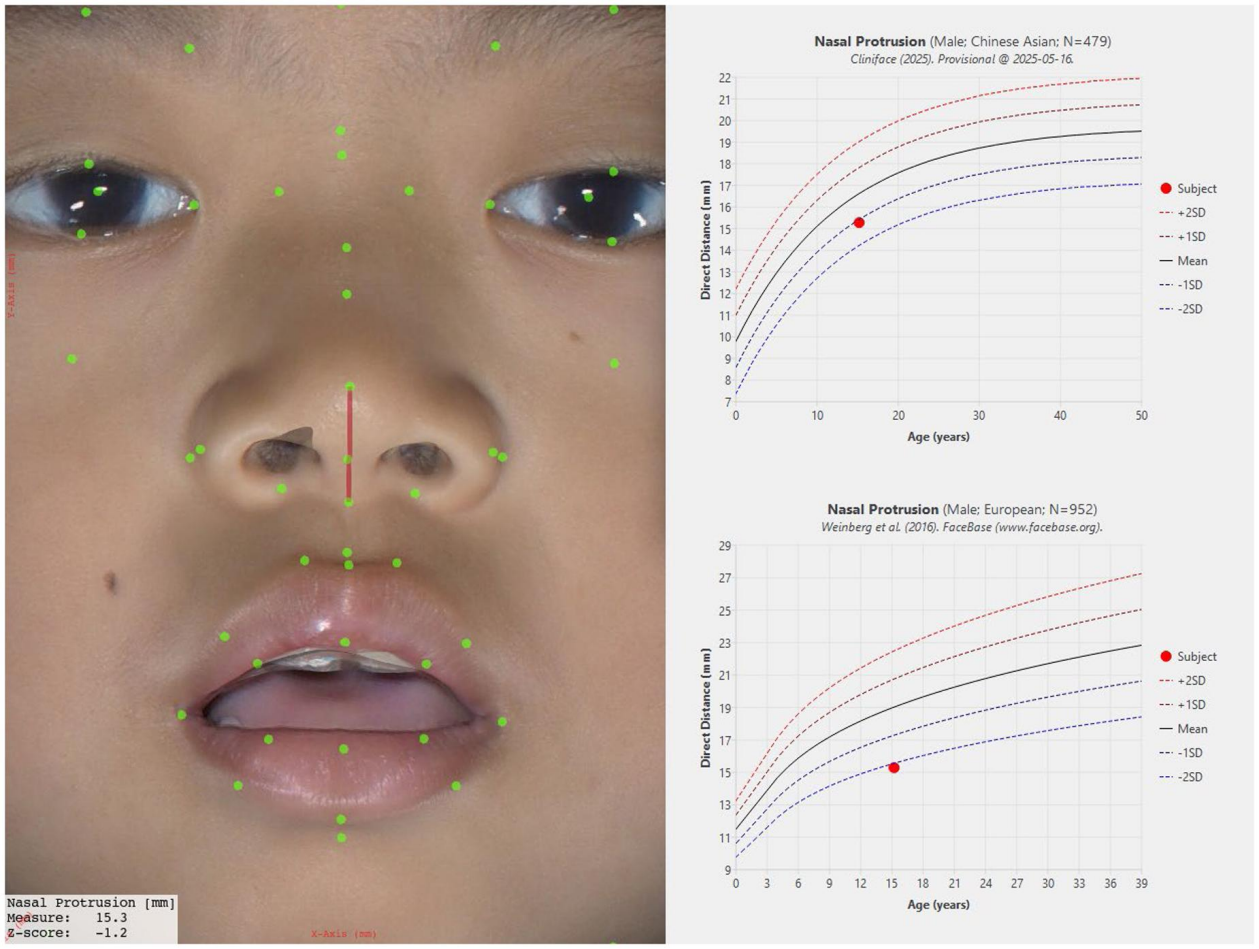
Composite image from cliniface showing how a selected 3D facial measurement (nasal protrusion) from a single individual is assessed against two different reference growth curves (image used with subject's parental permission).

To illustrate how such differences are highlighted within Cliniface, Figure 13 shows a composite of Cliniface's main 3D image window showing the facial landmarks with a selected measurement (Nasal Protrusion in this case) to the left of corresponding growth curves generated by Cliniface from its database. These charts inform the user how the measurement compares to the sex-matched Chinese reference (top chart) and the European reference (28) (bottom chart) for the same measurement. In this individual's case, the measurement is found to be within 2 standard deviations of the mean for their age on the Chinese reference (the exact Z-score is shown as −1.2 on the bottom left of the main image). The measurement is less than 2 SD when compared to the European statistics. The 2 SD threshold above and below the mean bounds approximately 95% of individuals within that demographic group. Depending on the specific criteria used, a measurement that sits outside of the ±2 SD threshold may contribute to Cliniface's detection of an atypical phenotypic trait.

**Figure 14:**
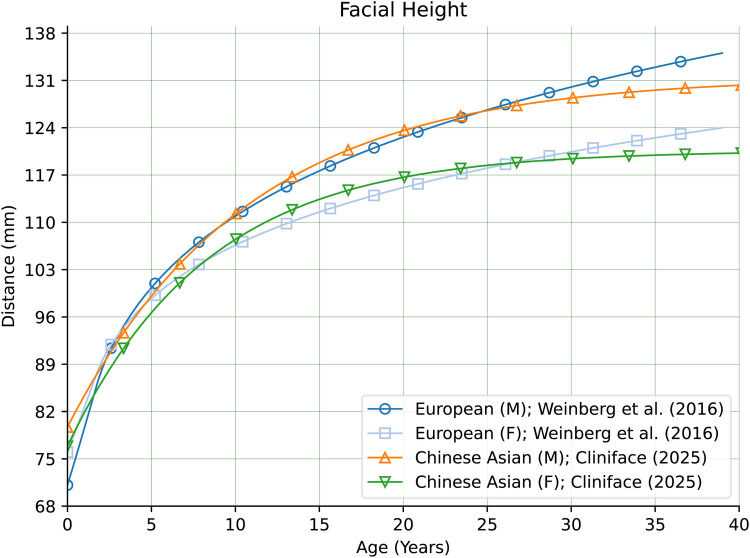
Facial height in males and females of Chinese vs. European genetic ancestry.

**Figure 15:**
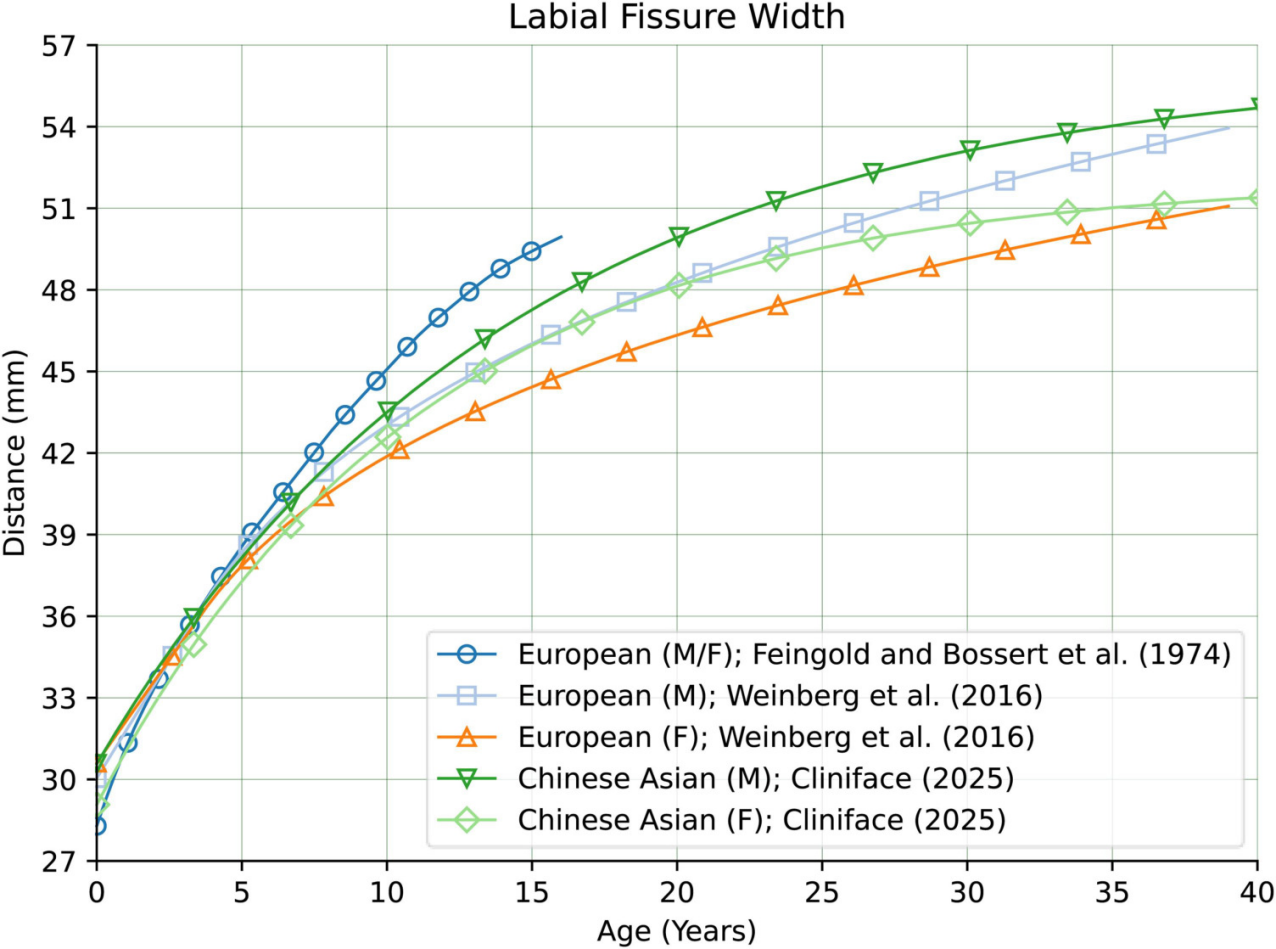
Labial fissure width in males and females of Chinese vs. European genetic ancestry.

Not all measurements were found to be so clearly different. Figure 14 shows clear sex-matched similarity across nearly the entire age range (with slight deviation within the first two years) of Facial Height between our Chinese reference cohort, and individuals of European genetic ancestry (28). Figure 15 shows how the Labial Fissure Width measurement (28, 35) is also very similar across the different groups.

**Figure 16:**
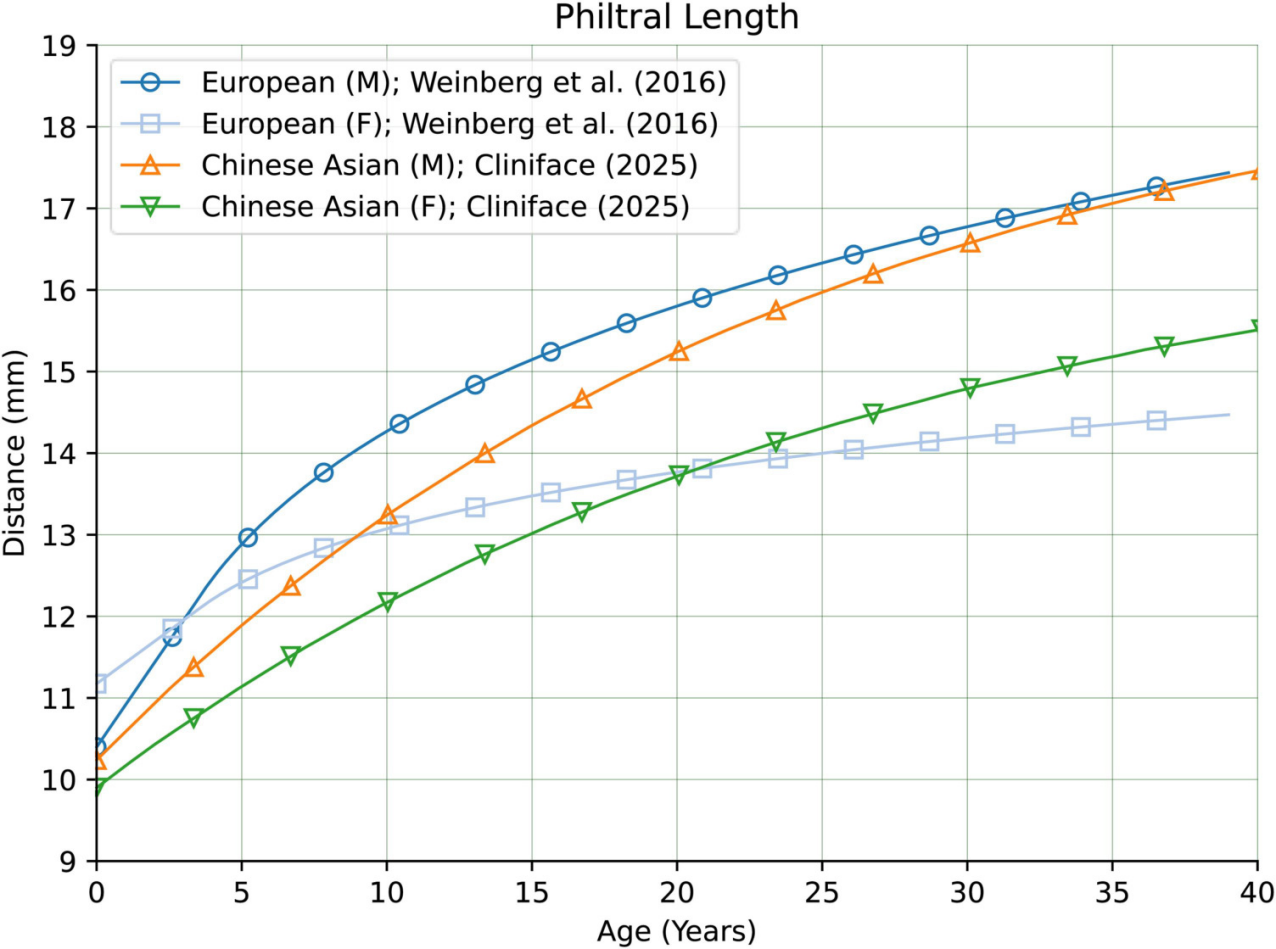
Philtral length in males and females of Chinese vs. European genetic ancestry.

As seen in Figure 16, Philtral Length was also very similar in magnitude overall between our Chinese reference group and individuals of European genetic ancestry (28). However, this measurement exhibited marked differences in growth rates between the two groups through childhood and adolescence.

## 4 Discussion

Our approach to conducting genetic ancestry-specific 3D facial analysis attempts to create standardization and differentiation for each group. Subtle and distinct facial differentiations can be identified in different genetic ancestry groups and subgroups with our methodology. Qiao et al. analyzed 2,379 Han Chinese in 15 categories after considering 14,838 facial traits with computer-aided specific facial phenotyping and a 3D manual landmarking database. Homogeneous and heterogeneous facial morphological traits were found amongst Han Chinese populations hailing from three geographical regions—Zhengzhou, Taizhou, and Nanning. 1,560 shared features were identified, defining the Han Chinese's basic facial morphology. While heterogeneous phenotypes corresponded to geographical subpopulations. The angle of the glabella, left subalare, and right cheilion were identified in geographical populations as the most significant facial variation (*p* = 3.4 × 10^−161^) (36). Individuals from Chinese (206 healthy adults) and North American Caucasian (206 healthy adults) genetic ancestry were compared to identify differences in horizontal, neoclassical facial canons by Dawei et al. Nose width in 51.5% of the Chinese genetic ancestry cohort corresponded to one-quarter of the face width (the nasofacial canon) as compared to 36.9% in the Caucasian genetic ancestry cohort. 38.8% of North American Caucasians had noses narrower than one-quarter of the face as opposed to 21.8% of Chinese, which was a statistically significant difference (37). Celebi et al. compared the facial morphology by gender between sexual dimorphism for facial features within Italian and Egyptian genetic ancestries. Both groups were found to have sexual dimorphism for facial features, and facial morphology was significantly different in both groups, specifically within females (38). Multi-ethnic Chicago Face Database (CFD) and the LACOP Face Database, comprising Brazilian subjects, were compared for facial asymmetry-based morphometric differences. The two databases found genetic ancestry groups-based differences in facial asymmetry (eye and mouth asymmetry). Creating multi-genetic ancestry facial databases as an essential prerequisite is reinforced by these asymmetry-based morphometric differences among databases and genetic ancestries validated in this study (39).

FaceBase consortium aims to create a nonnative 3D facial and genetic data infrastructure to utilize this resource to identify genes that impact normal midfacial differences. The first step involves a repertoire of 3D facial images and DNA samples from 3,500 healthy Caucasian individuals (age 5–40). Quantitative facial measurements will be derived from 3D images and genotyped using genome-wide SNP markers from DNA samples. To facilitate novel research approaches, the FaceBase repository aims to create a scalable, interactive and minable open data resource (40). Hallgrímsson et al. evaluated 3 D facial images of 7,057 individuals (3,327 with 396 varied syndromes), 727 of their relatives, and 3,003 unconnected, naive individuals to obtain syndrome diagnosis through machine learning approaches. Unconnected, naive individuals were identified with 96% exactitude, and the mixed group (syndromic and unconnected) definiteness was 73%. When the unconnected group was excluded, the syndrome's predictability increased to 78.1%. Phenotypic severity and facial distinctness were the predictors of categorization accuracy (41). Claes et al. deployed spatially dense quasi-landmarks to estimate facial shapes in mixed West African and European genetic ancestries from three locations (United States, Brazil, and Cape Verde). 3D facial images were mapped by a spatially dense mesh of 7,150 quasi-landmarks. Gender, genomic ancestry, and genotype, when modelled together, revealed the independent effects of selective alleles on facial features. A set of 20 genes had a marked impact on facial features, providing a unique mechanism for distinguishing genes that influence normal-range facial features and reflecting genetic markers of facial appearance (42).

Our premise behind conducting PI for different genetic ancestry groups is to assemble precise and dependable phenotypic data and apply these to assess disease susceptibility, diagnosis, treatment, prognosis, and prevention (43, 44). The complex phenotypic data can be visualized with the help of dynamic software such as Cliniface, thereby helping draw inferences between macro and micro-phenotypes. We also believe this is only the starting point, and we must create and build larger datasets involving more diverse genetic ancestry groups.

PI-based studies possess unique advantages to realize the true potential of precision medicine because they can unearth several risk factors, diagnostic markers, or prognostic predictors by correlating phenomic parameters with disease incidence or progression in different life stages of an individual or patient and also gender-specific anomalies (30–32, 45–47). Facial dysmorphology is a distinct feature of lysosomal storage disorders (LSDs), such as in the mucopolysaccharidoses and oligosaccharidosis (48). Similarly, dense surface models can be helpful for PI analysis of surface-based shape differences in rare diseases (RD) such Cornelia de Lange syndrome (49) and for Noonan, velo-cardio-facial, Smith–Magenis and Williams syndromes with discrimination rates ranging between 85% and 95% (50–52). Assimilating data from diverse sources, including phenomics, genomics, and multi-omics, provides valuable insights into disease etiologies, accurate diagnostics, and pathogenesis, and supports precision health (53).

We hope PI applications can establish distinctive and identifiable diagnostic biomarkers with simplified methodologies applicable in high and, low, and middle-income countries (LMICs). The advantages are manifold when compared to traditional approaches. These are—phenomic parameters are analyzed by artificial intelligence (AI), enhancing the efficiency, applicability, and scalability in identifying new diagnostic biomarkers; an image-driven approach helps establish the strength of the relationship between diagnostic biomarkers, clinical and phenomic parameters; a non-invasive approach cuts diagnosis time, especially in cases of RDs and reduces cost burden on the healthcare system. Global collaborations, open-access facial databases, and representation for diverse patient communities, with appropriate AI ethical guardrails, could help realize these possibilities. Strong adherence to legal frameworks, data governance, rigorous evaluation of data and privacy protection frameworks, and the elimination of algorithmic biases are key when PI intersects with biometric identification. A vast number of patients and individuals from LMICs remain unrepresented, and health equity can be attained only when these groups find representation in proportionate numbers in various studies. The author group intends to utilise this study as a stepping stone to conducting further studies on RD patients, including patients of non-Chinese genetic ancestries.

### 4.1 Shortcomings

Our study has several shortcomings, which we intend to highlight and use for course correction in our PI projects in the future. We conveniently sampled individuals of Chinese genetic ancestry living in Singapore with an emphasis on pediatric age groups. We could not include other genetic ancestry groups in the scope of this study because the sample size and resultant PI data were not statistically significant. The Chinese genetic ancestry group does display age bias, but this was unintentional due to the nature of the sampling method. The existing datasets are limited in scope, scale of sample size, and age distribution. Due to the bias in age sampling, we used a whole dataset approach to estimating growth in each of the measurements. The strength of this approach was that it allowed model parameters to be more tightly constrained. However, its weakness is in its inability to model different stages of growth independently. Finally, we reported only preliminary findings from a small number of individuals concerning how the newly generated Chinese reference range introduced herein caused the number and type of atypical phenotypic traits detected by Cliniface to change. This aspect requires a more complete investigation.

## 5 Conclusion

To achieve a more equitable impact of precision medicine, it is crucial to enhance the diversity of populations represented in genetic databases. Additionally, evaluating genetic scores alongside other disease-related factors will help ensure that precision medicine benefits all demographics. This approach promotes a more comprehensive understanding of how genetic variations interact with environmental and lifestyle influences, ultimately leading to more tailored and effective healthcare solutions for diverse patient populations (54). PI can evolve as one of the crucial areas for the further evolution of precision medicine. It is anticipated that combining multi-faceted PI data with genomic and clinical data aptly supported by AI can be instrumental in becoming a catalyst to establishing the diagnosis in data-deficient disease areas such as RDs but also be able to unravel phenomic parameters that may be interrelated with varying degrees of clinical outcomes associated with therapeutic interventions. Precision therapies can be developed and offered to eligible patients based on the evoked response. Our quest to translate that promise has started with putting together PI datasets for the genetic ancestry groups that historically lack representation and inclusion.

## Disclosure statement

SSJ is supported by the National Medical Research Council Clinician Scientist Award (NMRC/CSAINVJun21-0003). The rest of the authors have declared that no competing interests exist. Stuart Lee is an employee of Takeda Pharmaceuticals (Asia Pacific) Pte Ltd. Zi Qiang Teo was an employee of Takeda Pharmaceuticals (Asia Pacific) Pte Ltd at the time of the study (currently employed by GSK) and holds shares in Takeda Pharmaceuticals Company Limited. Consent: Written informed consent was obtained from the minor(s)' legal guardian for the publication of any potentially identifiable images or data included in this article (Figures 3, 13).

## Data Availability

The datasets presented in this article are not readily available because Data availability: No. Patient-level data on which the analysis was based will not be shared to protect patient anonymity. Requests to access the datasets should be directed to gareth.baynam@health.wa.gov.au.
