# Supplementary material for: Fostering equity in precision health through diverse 3D facial data
Source: Front Med Technol. 2026 Feb 19;8:1717535. doi: 10.3389/fmedt.2026.1717535 (PMC12960132; doi:10.3389/fmedt.2026.1717535)
Supplement: Supplementary file 1 [file Table1.docx]

Chinese genetic ancestry distribution across age groups

| **Chinese** |  |  |  |
| --- | --- | --- | --- |
| **Age** | **Female** | **Male** | **Sum** |
| 0 | 6 | 9 | 15 |
| 1 | 12 | 20 | 32 |
| 2 | 11 | 16 | 27 |
| 3 | 22 | 16 | 38 |
| 4 | 20 | 28 | 48 |
| 5 | 27 | 24 | 51 |
| 6 | 30 | 36 | 66 |
| 7 | 37 | 49 | 86 |
| 8 | 27 | 43 | 70 |
| 9 | 22 | 35 | 57 |
| 10 | 28 | 41 | 69 |
| 11 | 18 | 40 | 58 |
| 12 | 4 | 21 | 25 |
| 13 | 11 | 19 | 30 |
| 14 | 17 | 13 | 30 |
| 15 | 6 | 14 | 20 |
| 16 | 12 | 12 | 24 |
| 17 | 4 | 8 | 12 |
| 18 | 1 | 1 | 2 |
| 19 | 0 | 0 | 0 |
| 20 | 0 | 0 | 0 |
| 21 | 0 | 0 | 0 |
| 22 | 2 | 0 | 2 |
| 23 | 0 | 0 | 0 |
| 24 | 2 | 1 | 3 |
| 25 | 0 | 0 | 0 |
| 26 | 1 | 0 | 1 |
| 27 | 1 | 0 | 1 |
| 28 | 0 | 0 | 0 |
| 29 | 3 | 0 | 3 |
| 30 | 1 | 0 | 1 |
| 31 | 2 | 2 | 4 |
| 32 | 3 | 3 | 6 |
| 33 | 2 | 0 | 2 |
| 34 | 4 | 1 | 5 |
| 35 | 4 | 2 | 6 |
| 36 | 2 | 0 | 2 |
| 37 | 7 | 4 | 11 |
| 38 | 1 | 1 | 2 |
| 39 | 9 | 3 | 12 |
| 40 | 1 | 1 | 2 |
| 41 | 8 | 2 | 10 |
| 42 | 5 | 1 | 6 |
| 43 | 3 | 3 | 6 |
| 44 | 0 | 0 | 0 |
| 45 | 4 | 0 | 4 |
| 46 | 6 | 4 | 10 |
| 47 | 5 | 1 | 6 |
| 48 | 1 | 1 | 2 |
| 49 | 5 | 0 | 5 |
| 50 | 0 | 1 | 1 |
| 51 | 2 | 2 | 4 |
| 52 | 0 | 0 | 0 |
| 53 | 0 | 0 | 0 |
| 54 | 0 | 1 | 1 |
| 55 | 0 | 0 | 0 |
| 56 | 0 | 0 | 0 |
| 57 | 0 | 0 | 0 |
| 58 | 0 | 0 | 0 |
| 59 | 0 | 0 | 0 |
| 60 | 0 | 0 | 0 |
| 61 | 0 | 0 | 0 |
| 62 | 0 | 0 | 0 |
| 63 | 0 | 0 | 0 |
| 64 | 0 | 0 | 0 |
| 65 | 0 | 0 | 0 |
| 66 | 0 | 0 | 0 |
| 67 | 0 | 0 | 0 |
| 68 | 0 | 0 | 0 |
| 69 | 1 | 0 | 1 |
| **TOTAL** | **400** | **479** | **879** |

Atypical Traits identified by Cliniface in a small sample of individuals before and after introduction of the Chinese reference range

| **Genetic Ancestry** | **Before** | | **After** | |
| --- | --- | --- | --- | --- |
|  | **Identified Phenotypic Traits** | **Count** | **Identified Phenotypic Traits** | **Count** |
| Chinese | HP:0005280 Nasal Bridge, Depressed HP:0012745 Palpebral Fissures, Short HP:0012802 Jaw, Wide | 3 | HP:0012745 Palpebral Fissures, Short | 1 |
| Chinese | HP:0000283 Face, Broad HP:0000337 Forehead, Broad HP:0000431 Nasal Bridge, Wide HP:0000445 Nose, Wide HP:0000506 Telecanthus HP:0000508 Ptosis HP:0005280 Nasal Bridge, Depressed HP:0011800 Midface retrusion HP:0012802 Jaw, Wide | 9 | HP:0000283 Face, Broad HP:0000445 Nose, Wide HP:0012802 Jaw, Wide | 3 |
| Chinese | HP:0000283 Face, Broad HP:0000337 Forehead, Broad HP:0000506 Telecanthus HP:0002000 Columella, Short HP:0012802 Jaw, Wide | 5 | - | 0 |
| Chinese | HP:0000289 Philtrum, Broad HP:0000337 Forehead, Broad HP:0000506 Telecanthus HP:0000520 Proptosis HP:0012371 Hyperplasia of midface | 5 | HP:0000289 Philtrum, Broad HP:0000520 Proptosis | 2 |
| Chinese | HP:0000283 Face, Broad HP:0000337 Forehead, Broad HP:0000506 Telecanthus HP:0002000 Columella, Short HP:0005280 Nasal Bridge, Depressed HP:0012802 Jaw, Wide | 6 | - | 0 |
| Chinese | HP:0000337 Forehead, Broad HP:0000463 Anteverted nares HP:0000506 Telecanthus | 3 | HP:0000219 Upper Lip Vermilion, Thin | 1 |
| Chinese | HP:0000215 Upper Lip Vermilion, Thick HP:0000283 Face, Broad HP:0000490 Eyes, Deeply Set HP:0000508 Ptosis HP:0002000 Columella, Short HP:0005280 Nasal Bridge, Depressed HP:0012371 Hyperplasia of midface HP:0012802 Jaw, Wide | 8 | - | 0 |
| Chinese | HP:0000215 Upper Lip Vermilion, Thick HP:0000283 Face, Broad HP:0000337 Forehead, Broad HP:0000506 Telecanthus HP:0002000 Columella, Short HP:0005280 Nasal Bridge, Depressed HP:0012802 Jaw, Wide | 7 | HP:0002000 Columella, Short | 1 |
| Malay | HP:0000337 Forehead, Broad HP:0000520 Proptosis HP:0002000 Columella, Short | 3 | HP:0000520 Proptosis | 1 |
| Malay | HP:0000337 Forehead, Broad HP:0002000 Columella, Short | 2 | - | 0 |
| Filipino | HP:0000283 Face, Broad HP:0000337 Forehead, Broad HP:0002000 Columella, Short HP:0012802 Jaw, Wide | 4 | - | 0 |
| Indian | HP:0000494 Palpebral Fissure, Downslanted HP:0000520 Proptosis HP:0012371 Hyperplasia of midface | 3 | HP:0000426 Nasal Bridge, Prominent HP:0000494 Palpebral Fissure, Downslanted HP:0000520 Proptosis | 3 |
| Pakistani | HP:0000311 Face, Round HP:0000337 Forehead, Broad HP:0000426 Nasal Bridge, Prominent HP:0000520 Proptosis HP:0012371 Hyperplasia of midface | 5 | HP:0000311 Face, Round HP:0000426 Nasal Bridge, Prominent HP:0000460 Nose, Narrow HP:0000520 Proptosis HP:0000637 Palpebral Fissures, Long HP:0012371 Hyperplasia of midface | 6 |
